# Collaboration Around Technology Using Community Health Workers in Type 1 Diabetes (CATCH‐T1D): Protocol and Preliminary Results for a Randomized Controlled Trial

**DOI:** 10.1155/jdr/8785953

**Published:** 2026-04-08

**Authors:** Shivani Agarwal, Molly Finnan, Kevin P. Fiori, Ashby Walker, Clyde Schechter, Renee Whiskey, Andrew Telzak, Charlotte Chen, Jovan Milosavljevic, Astou Talla, Steven Sanchez, Priyanka Mathias, Judith A. Long

**Affiliations:** ^1^ Fleischer Institute of Diabetes and Metabolism, Montefiore Medical Center-Albert Einstein College of Medicine, Bronx, New York, USA; ^2^ New York Regional Center for Diabetes Translation Research, Albert Einstein College of Medicine, Bronx, New York, USA, yu.edu; ^3^ Department of Family and Social Medicine, Albert Einstein College of Medicine, Bronx, New York, USA, yu.edu; ^4^ Community Health Worker Institute, Albert Einstein College of Medicine, Bronx, New York, USA, yu.edu; ^5^ Department of Pediatrics (Academic General Pediatrics), Albert Einstein College of Medicine, Bronx, New York, USA, yu.edu; ^6^ Department of Health Services Research, Management and Policy, University of Florida, Gainesville, Florida, USA, ufl.edu; ^7^ Department of Pediatrics (Pediatric Endocrinology & Diabetes), Montefiore Medical Center, Bronx, New York, USA, montefiore.org; ^8^ Division of General Internal Medicine, Perelman School of Medicine, University of Pennsylvania, Philadelphia, Pennsylvania, USA, upenn.edu; ^9^ Department of Veteran Affairs, Corporal Michael J. Crescenz VA Medical Center, Philadelphia, Pennsylvania, USA, va.gov

**Keywords:** community health worker, diabetes, diabetes technology, disparities, ethnicity, inequity, minority, race, Type 1 diabetes, young adult

## Abstract

**Background:**

Diabetes technology use in Type 1 diabetes (T1D) is the standard of care given broad biological and psychosocial benefits. Hispanic and Non‐Hispanic Black young adults are the fastest‐growing demographic with T1D in the United States and face inequity in technology use, leading to poor outcomes. We explored whether community health workers (CHWs) could coaddress unmet social needs and navigation of T1D technology to improve standard of care and outcomes in this high‐risk population.

**Methods:**

The collaboration around technology using community health workers in Type 1 diabetes (CATCH‐T1D) intervention enhances the CHW model by integrating social needs management with T1D technology navigation. CHWs trained at the Montefiore‐Einstein CHW Institute navigate YA with T1D to technology by offering information access, shared decision‐making, and health system–pharmacy coordination. We are performing an ongoing trial testing CATCH‐T1D against usual care using a 9‐month hybrid effectiveness–implementation design for 130 Hispanic and/or non‐Hispanic Black young adults (18–35 years) with T1D at Montefiore Medical Center in the Bronx.

**Results:**

One hundred and eighteen participants have been randomized (48% F, 67% Hispanic, 72% Medicaid, mean HbA1c: 9.2%). Thus far, CHWs have conducted 476 follow‐up encounters and resolved 24% of identified health‐related social needs. Postintervention interviews showed high participant satisfaction and increased engagement with diabetes self‐management due to CHWs′ flexible communication, support with diabetes technology, and assistance with social needs, fostering trust in the healthcare system.

**Discussion:**

CATCH‐T1D is a specialized CHW model that simultaneously addresses social needs and navigation to diabetes technology, with potential to provide a new scalable model of care.

## 1. Introduction

Growing evidence from our group and others have identified major inequity in outcomes between Non‐Hispanic Black and Hispanic versus White young adults (YAs) with Type 1 diabetes (T1D), noting consistent glycemic disparities with 2.3% higher HbA1c values, 2–3 times higher rates of hospitalizations for diabetic ketoacidosis (DKA) and hypoglycemia, and 1.5–2 times higher risk of premature mortality [1–4]. In recent years, there have been tremendous advancements in the field of diabetes technology to improve T1D clinical outcomes. Use of continuous glucose monitors (CGMs) and automated insulin delivery (AID) systems is now recommended as standard of care among people with T1D by the American Diabetes Association (ADA) and other professional societies [2–4]. Nevertheless, despite established benefits and increasing insurance coverage of T1D technologies, widespread racial‐ethnic inequities in use have persisted, which predicts long‐term poor clinical outcomes [4].

Evidence suggests that inequity in T1D technology use stems from multilevel barriers, including not being universally offered technology by providers, lack of shared decision‐making, difficulties navigating insurance, and difficulty troubleshooting technology failures and connectivity [2–5]. Endocrinology and primary care providers cite challenges due to limited time and resources for patient technology education and follow‐up outreach, alongside their own limited knowledge surrounding T1D technology use [3–5]. Previous interventions have focused on solutions tailored to expanding insurance coverage and lowering overall cost of diabetes technology. However, these interventions have not been sufficient in bridging inequities in technology use [5–7]. Even in healthcare systems where universal coverage for technology is offered, differences in technology adoption persists. Our pivotal studies in minoritized YA with T1D showed that health‐related social needs (HRSNs) predicted use of diabetes technology use, which were major contributors to the HbA1c level disparity between Hispanic and Non‐Hispanic Black versus White YA [5, 8]. Multilevel interventions are critical to prevent further inequity from expanding in this high‐risk population [1, 8].

Community health workers (CHWs) are trusted members from the communities in which they serve, providing social needs screening and management, community outreach, and healthcare system navigation [7, 9, 10]. CHW interventions in Type 2 diabetes have successfully demonstrated improvement in diabetes and general health outcomes by providing extra outreach and social needs support [11–16]. Additional studies also show promising results of CHW interventions in improving mental health and quality of care, as well as reducing disparities [14–17]. While CHW interventions have a strong evidence base of effectiveness in reducing inequity among minoritized populations with diabetes, their potential to increase T1D technology use has not yet been realized. A specialized T1D CHW model could address barriers to offering information access from a trustworthy source, employing shared decision‐making in decisions regarding technology use, navigating the often confusing and cumbersome process of obtaining and using T1D technology, and addressing competing unmet social needs.

The collaboration around technology using community health workers in Type 1 diabetes (CATCH‐T1D) intervention is a new specialty care CHW model that integrates social needs management and navigation to T1D technologies. CATCH‐T1D is aimed at building upon the existing success of CHW models in chronic disease and adapts for T1D‐specific needs.

**Figure 1:**
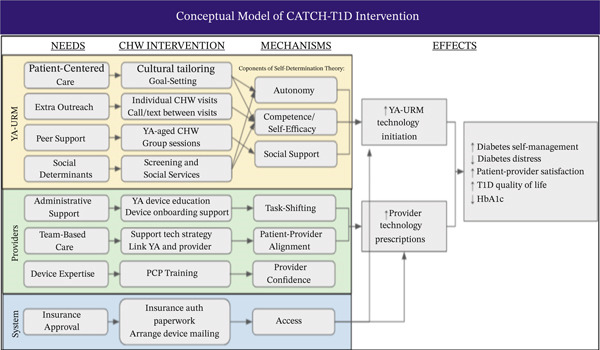
Conceptual model of CATCH‐T1D intervention.

The conceptual model of the CATCH‐T1D intervention was developed based on the theory of self‐determination [18], as well as extensive mixed‐methods evaluation by our group [2, 4, 5, 8]. The theory of self‐determination describes how the three components of autonomy, competence, and social support increase self‐determination and self‐care behaviors. This theory has been studied in adolescents with diabetes, with increased self‐determination shown to improve blood glucose monitoring, medication‐taking, and adherence to diet and physical activity [18]. By mapping the CATCH‐T1D intervention onto the theory of self‐determination, we designed the components of CATCH‐T1D to increase autonomy, competence, and social support of underrepresented YA with T1D, with the aim of increasing self‐determination and improving diabetes self‐management and clinical outcomes (Figure 1).

We designed CATCH‐T1D to address YA, provider, and system needs, identified through our prior research [4, 5, 8]. This includes enhancing shared decision‐making, providing device education and support while onboarding technology, addressing prior authorization and insurance navigation challenges related to technology adoption, and aiding patients and providers in troubleshooting technology failures and connectivity issues. The specific aims are as follows:

Aim 1. Evaluate CATCH‐T1D effects on technology initiation and continued use.

Hypothesis: Compared with usual care, CATCH‐T1D will significantly increase technology initiation and continued use at 9 months (primary: % initiation; secondary: % of initiated use at 9 months). We will measure potential mediators and distal health outcomes to inform preliminary efficacy and future effect sizes.

Aim 2. Evaluate CATCH‐T1D implementation using Proctor′s taxonomy of implementation outcomes: Implementation science methods will guide the evaluation of acceptability, feasibility, adoption, fidelity, and cost to inform future larger pragmatic trials and overall sustainability.

## 2. Materials and Methods

### 2.1. Study Design

**Figure 2:**
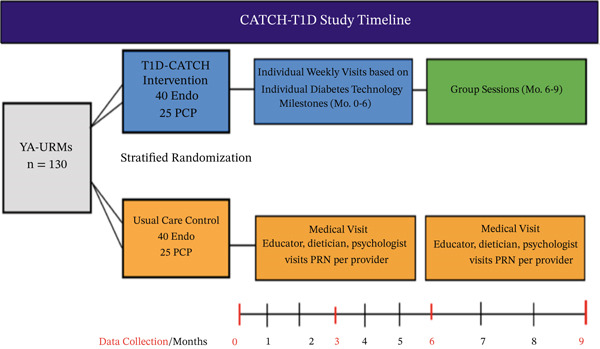
CATCH‐T1D study timeline.

CATCH‐T1D is being tested in an ongoing randomized controlled trial against usual care, using a Type 1 effectiveness–implementation hybrid design. Outcome assessments are being conducted at 3, 6, and 9‐month timepoints after randomization. Intervention arm participants work with CHWs, virtually or in‐person, on an individual basis for 6 months. From months 6 to 9, study arm participants can attend optional CHW‐led group sessions (Figure 2).

Group sessions give intervention arm participants the opportunity to meet and discuss their experiences on diabetes technology with other participants. These sessions are also used as a space for participants who are still hesitant about starting diabetes technology to learn from participants who have started using technology.

### 2.2. Qualitative Analysis

All intervention participants were invited to complete an interview upon reaching the 6‐month point in the study. Interview guides covered the following topics: (1) intervention feasibility and acceptability, (2) care coordination and outreach methods, (3) diabetes technology support, (4) peer and emotional support, (5) self‐efficacy, (6) social needs navigation, and (7) YA‐centered care. Interviews were conducted telephonically by a member of the study team.

Interview recordings were transcribed by a third‐party transcription service, uploaded to NVivo, and analyzed by two independent coders using an inductive coding approach until thematic saturation was reached. When new codes emerged, the coding framework was revised, and transcripts were reanalyzed accordingly. Through this process, categories were identified, refined, and organized into broader themes.

Coding discrepancies were resolved through documentation, discussion among coders, and consultation with the study team. These discussions informed data interpretation and contributed to refinements in the analytic process. An audit trail—including recordings, transcripts, and meeting notes—was maintained to document how data were generated, analyzed, and interpreted.

### 2.3. Eligibility Criteria

Inclusion criteria are as follows: (1) T1D duration ≥ 6 months, (2) 18–35 years old, (3) self‐identified non‐Hispanic Black or Hispanic race‐ethnicity, (4) English‐ or Spanish‐speaking, and (5) not currently on a connected diabetes technology system (includes never offered, prescribed but not started within 3 months of receiving the device, discontinued, or previously refused technology).

Exclusion criteria are as follows: (1) developmental or sensory disability interfering with study participation, (2) current pregnancy, and (3) participation in another behavioral or technology intervention study in the past 6 months.

### 2.4. Participant Recruitment and Follow‐Up

Recruitment staff conduct weekly medical record reviews to compile lists of potentially eligible patients from Montefiore adult and pediatric endocrinology practices, primary care clinics, and hospital/ambulatory sites. Recruitment and consent are done in person or by phone to reduce selection bias of patients who come to clinic appointments. A baseline assessment is performed via REDCap, after which participants are randomized 1:1 to either the CATCH‐T1D intervention or usual care. Self‐reported survey data is collected at 3, 6, and 9 months via text, email, or over the phone per participants′ preference. Recruitment and retention procedures are guided by our prior experience for best practices when working with YA with T1D [4, 5, 19, 20].

### 2.5. CATCH‐T1D Intervention Arm

#### 2.5.1. CATCH‐T1D CHW Role

The CATCH‐T1D model was developed through a series of user‐centered design workshops that engaged a multidisciplinary panel of stakeholders, including YA with T1D, endocrinology and primary care providers, and CHWs from the Bronx, New York. These workshops were aimed at generating ideas and proposing actionable strategies to reduce disparities in diabetes technology use among individuals with T1D [21].

Feedback from these sessions highlighted the importance of peer involvement and additional support from healthcare teams as key factors in enhancing the acceptance and utilization of diabetes technology among YA with T1D. Moreover, CHWs identified themselves as particularly effective in these interventions due to their deep community and cultural connections, as well as their capacity to provide more extensive patient outreach and social support [21].

**Figure 3:**
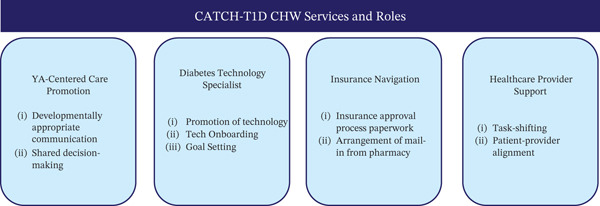
CATCH‐T1D CHW services and roles.

Thus, CATCH‐T1D enhances the CHW model by augmenting core roles of patient advocacy, outreach, coaching, education, care navigation, and social service linkage [11] with specific training on diabetes technology. CATCH‐T1D CHWs provide technology education, assistance with goal setting, peer support, and social needs management, while shifting insurance approval work from providers and helping to better align patient–provider priorities (Figure 3).

#### 2.5.2. CATCH‐T1D CHW Training

CATCH‐T1D CHW training builds upon the foundation of the Montefiore Community Health Worker Institute (CHWI) model, using their services for recruitment, training, oversight, and continued support [13, 22, 23]. The CHWI facilitates and uses best‐practice approaches to CHW recruitment, training, deployment, ongoing role development, and integration into health systems, while creating a sustainable, cost‐effective, value‐based model [13, 22, 23]. Montefiore has 22 integrated CHWs in ambulatory practices and federally qualified health centers, where CHWs work and communicate directly with providers. Montefiore CHWs are trained in core roles aligned with national guidelines [11] and are overseen jointly by a successful CHW supervisor–health system leader model [13, 21, 22]. CHWs are hired from the hospital system′s catchment area within the Bronx, sharing lived experience with our patients, and are credentialed through New York state and contracted with Montefiore Medical Center System, with Montefiore ID badges and electronic medical record viewing and note‐writing access.

**Figure 4:**
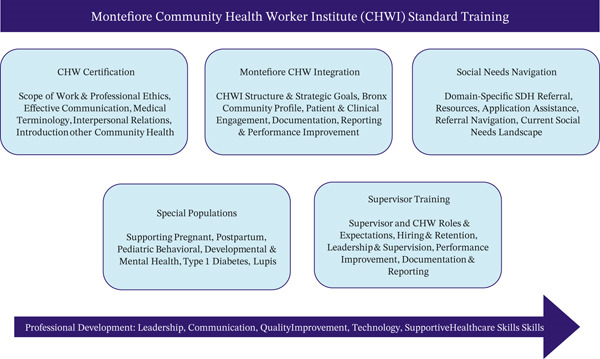
Montefiore Community Health Worker Institute (CHWI) standard training.

For CATCH‐T1D CHWs, standard training was provided by CHWI, which combines robust orientation to the CHW role at Montefiore Einstein and adapted to varied learning styles including facilitated and self‐guided web‐based and in‐person sessions, assigned readings, self‐paced learning assignments and exploration, and scenario‐based role‐playing and group work. Social service navigation training focuses intensely on laying the foundation for social needs navigation of each social service domain: housing security, housing quality, food security, utilities, transportation, access to healthcare, child/adult care, and legal services. Facilitated training covers the current social needs landscape for each domain, common social needs services, domain‐specific social needs algorithms, recommended resources, advocating for patients, and teaching them how to advocate for themselves (Figure 4).

**Figure 5:**
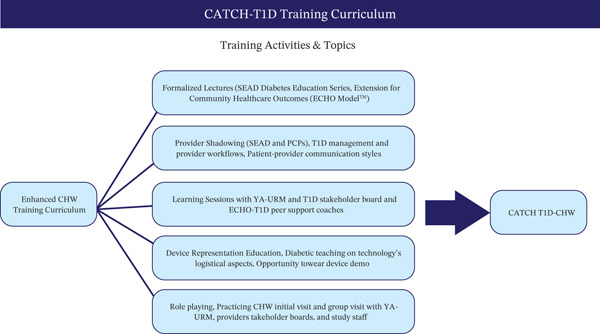
CATCH‐T1D training curriculum.

CATCH‐T1D additional T1D training is detailed in Figure 5, which included formalized lectures on the Supporting Emerging Adults with Diabetes (SEAD) education program [2, 11] and shadowing providers in SEAD and PCPs to familiarize themselves with provider workflows and communication styles. Additional training included CHW meeting with the YA‐URM with T1D stakeholder board to learn about their lived experience, participation in didactics on T1D technology devices led by device representatives, and role‐playing exercises to practice initial and group visits with the YA‐URM advisory board and study staff.

#### 2.5.3. CATCH‐T1D CHW Procedures

Participants complete an introductory visit—either in person or virtually with their CHW—during the first week after enrollment, based on their preference. Follow‐up visits occur weekly during the first month, biweekly during Months 2–3, and monthly during Months 4–6. CHW–participant interactions take place via secure video or phone calls or in person, and communication between scheduled visits is tailored to each participant′s needs and preferences.

During individual sessions, CHWs introduce participants to and familiarize them with available diabetes technology using technology demos and hands‐on demonstrations. They educate participants about different devices, explain their potential benefits, and help identify options that best align with each person′s preference and lifestyle. CHWs also offer practical guidance on how diabetes technology can support daily self‐management and empower participants to make informed decisions in collaboration with their diabetes care team. When a participant expresses interest in starting a technology, the CHW notifies the primary diabetes provider, facilitates insurance approval, and assists with the initiation process.

CHWs also play a critical role in identifying and addressing social needs that may affect diabetes management. They explore factors such as food insecurity, transportation challenges, difficulty accessing medical care or supplies, housing instability, financial strain, and gaps in insurance coverage. Through ongoing check‐ins, CHWs are trained to recognize barriers participants may or may not explicitly mention. Once a need is identified, they help participants navigate community resources and support programs, assisting with applications for financial or insurance support, coordinating transportation, and connecting individuals with social service organizations as appropriate. CHWs further support participants by helping them navigate the health system, including scheduling appointments and facilitating communication with their clinical team. Between sessions, participants are encouraged to reach out to their CHW with updates regarding diabetes concerns, technology questions, or emerging social needs.

**Figure 6:**
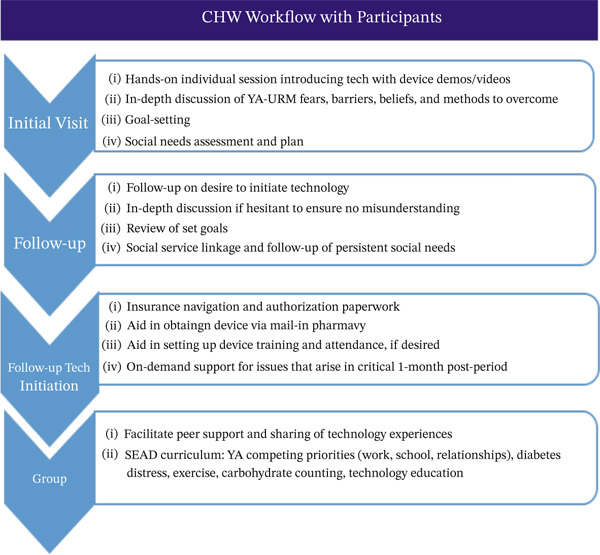
CHW workflow with participants. SEAD: Supporting Emerging Adults with Diabetes, ECHO: Extension for Community Healthcare Outcomes, PCPs: primary care practitioners, YA‐URM: young adults of underrepresented minority groups.

After the initial meeting with participants, CHWs contact each participant′s diabetes care providers to introduce themselves and discuss any concerns providers may have regarding the participant′s readiness for technology. CHWs continue to communicate with providers through the medical record at several key points during the study, including when (1) a participant expresses interest in starting technology, (2) insurance approval is obtained, (3) the participant receives and is trained on a device, (4) issues arise with technology or prescriptions, and (5) urgent diabetes‐related needs emerge. CHWs also help represent participant concerns in communications with providers to ensure alignment between participant goals and clinical priorities. They encourage participants to attend scheduled medical visits and to reach out to their providers when clinical questions or concerns arise. CHW workflow with participants is detailed in Figure 6.

#### 2.5.4. Usual Care Control Arm

Control arm participants receive usual primary or endocrine care at Montefiore, consisting of a physician or nurse practitioner visit. During these visits, review of blood sugars and treatment decisions based on provider experience and practice guidelines are employed. Primary care physicians share resources such as certified diabetes care and education specialists, chronic care registered nurses, nutritionists, licensed clinical social workers, and blood glucose data download capability but lack dedicated infrastructure or expertise in T1D or diabetes technology. Physicians in endocrinology practices are nested within a diabetes academic center with access to diabetes nurse practitioners, educators, dieticians, psychologists, and technology trained nurses. All practices are advised to adhere to ADA standards of care, including regular visits with their physician or nurse practitioner every 3 months and attending individual or group sessions with allied diabetes health professionals as required.

#### 2.5.5. Study Measures and Outcomes

Measurements are completed at baseline, 3, 6, and 9 months. The detailed outcome measures and their descriptions are outlined in Tables 1 and 2 and described below.

**Table 1:** Table of measures.

Domain	Source of data	Frequency of measurement	Description
*Aim 1. Effectiveness outcomes*			
Technology use *Primary*: Initiation *Secondary*: Continued use	Self‐reported (REDCap), EMR prescriptions, CHW dashboard, device cloud‐based platforms, and company technology use reports	Baseline, 3, 6, and 9 months	Technology yes/no: Any upgrade in technology included as part of the primary outcome: *No tech to CGM or pump, CGM to AID, disconnected CGM + pump to AID*
*Aim 2. Implementation outcomes*			
Feasibility	Postintervention interviews	6 months	Intervention content, complexity, comfort, delivery, credibility
Adoption	Recruitment logs, EMR	9 months	YA consent rates, % provider opt‐in, CHW communications
Fidelity	CHW dashboard, session recordings, EMR	3, 6, and 9 months	Session attendance, content delivery, insurance tasks
Cost	Time sheets, receipts, budget	9 months	CHW salary/benefits, consumables

**Table 2:** Exploratory outcomes and potential mediators of effect.

Health‐related social needs	EMR	Baseline, 3, 6, and 9 months	Housing security, housing quality, food security, utilities, transportation, access to healthcare, child/adult care, and legal services
Glycemia	EMR	Baseline, 3, 6, and 9 months	HbA1c, time in range, time above range, time below range, coefficient of variability (CV)
Healthcare utilization	EMR	Baseline, 3, 6, and 9 months	Office visits, ED visits, hospitalizations
Psychosocial health	Self‐report surveys	Baseline, 3, 6, and 9 months	Quality of life, diabetes distress, diabetes self‐management, satisfaction in care (provider and CHW measures)
Demographics	EMR, self‐report surveys	Baseline, 3, 6, and 9 months	Age, sex, race‐ethnicity, preferred language

For Aim 1, the primary outcome of technology use includes initiation of any technology by 9 months. Any upgrade in technology will be included as part of the primary outcome: no technology to any technology (CGM and/or pump), CGM to AID system, and disconnected CGM + pump to AID. The primary outcome is captured from a variety of sources including the EMR, reports from technology dashboards, training dates, and participant self‐report surveys. Confirmation of technology uptake and continuation is cross‐referenced with the associated device data platforms, when information for login to personal accounts is supplied by participants.

For Aim 2, implementation outcomes are being measured throughout the duration of the study to inform future larger pragmatic trials and overall sustainability. Brief postintervention interviews with participants explore feasibility domains of intervention content, complexity, comfort, delivery, and credibility. Adoption of the intervention is being measured using YA consent rates, number of providers who participate, and number/type of EMR‐documented CHW communications with YA‐URMs and providers. The CHW dashboard monitors intervention fidelity by tracking CHW activities. Costs are being collected from two broad categories: labor (FTE) and consumables (transportation, printed materials).

#### 2.5.6. Statistical Analysis Plan

For Aim 1, we will analyze primary and secondary outcomes using multilevel logistic models, incorporating the randomized treatment arm, all four measurement times, and the interaction between treatment arm and time. The coefficient of the interaction between treatment arm and each time is the difference‐in‐differences estimator of the treatment effect at that time, which is properly adjusted for baseline differences. The results will be presented as the interaction regression coefficients with 95% confidence intervals.

A small amount of data may be sporadically missing at random or completely at random due to failures in measurement or data collection systems. Missing questionnaire item responses may also be missing at random. These will be dealt with by multiple imputation or case‐wise deletion, respectively. Missing data due to subject withdrawal or nonresponse are generally missing not at random. These will be dealt with by sensitivity analyses to determine robustness of the results.

For Aim 2, adoption and fidelity will be summarized using descriptive statistics, showing counts and percent for categorical variables and means and standard deviations or medians and interquartile ranges for continuous variables, as appropriate. Acceptability will be measured through postintervention interviews. Two independent coders will use NVivo software and a standardized codebook based on the Proctor feasibility framework 24. For cost analyses, the research staff will calculate labor, durable equipment, and consumable resource use in the CHW arm, less that in the control arm, as the net cost of the intervention. Costs per participant, per participant who initiates technology, per participant with continued use, and per percentage point decrease in HbA1c will be calculated.

## 3. Results

To date, 128 participants have been approached for this study, from which 118 participants have been consented, enrolled, and randomized. Mean age is 24.4 years; the proportion female was 48% (*n* = 57); proportion insured by Medicaid is 72% (*n* = 85); mean HbA1c is 9.2%. Proportion Hispanic is 67% (*n* = 80) and non‐Hispanic Black is 32% (*n* = 38).

CATCH‐T1D CHWs have conducted 476 follow‐up encounters with *n* = 58 intervention participants with a mean of 9 visits per participant and range of 1–20 visits (in‐person: *n* = 22, Zoom: *n* = 191, telephone calls: *n* = 253, other: *n* = 10). In addition, documentation of 419 activity encounters was logged which included work of the CHW in between visits with participants on tasks such as diabetes technology navigation, care coordination, and social needs service linkage.

Of 115 participants who have completed baseline survey assessments, 56% (*n* = 64) reported at least one HRSN. Fear of food running out before the end of the month was the most reported social need (*n* = 34, 20%), followed by skipping purchasing medication or attending medical visits due to cost (*n* = 30, 18%), transportation needs (*n* = 29, 17%), housing quality (*n* = 22, 13%), stable housing (*n* = 20, 12%), utility needs (*n* = 13, 7%), legal help (*n* = 12, 7%), and child care (*n* = 6, 3%). For the 166 distinct HRSNs identified, 24% (*n* = 40) were documented as resolved within the 9‐month timeframe.

Semistructured, postintervention interviews were conducted with 21 participants. The mean age was 25 years; 57% were female (*n* = 12). Forty‐eight percent identified as Hispanic (*n* = 10) and 52% as non‐Hispanic Black (*n* = 11).

From postintervention interviews describing participants′ experiences with the CATCH‐T1D CHW, several common themes emerged:

• High satisfaction and increased engagement in diabetes self‐management, attributed to the flexibility in communication methods and scheduling that CHWs provided compared with traditional healthcare visits.
• CHWs as a valuable source of support, offering practical information about diabetes technology and serving as a consistent, accessible resource for addressing unmet social needs.
• Strengthened trust in the healthcare system, fostered through shared decision‐making with CHWs, which in turn increased participants′ willingness to consider and adopt diabetes technology.

## 4. Discussion

CATCH‐T1D is unique in its use of specialty care CHWs to be responsive to underserved YA with T1D who are struggling with unmet competing social needs, lack of T1D technology, and poor outcomes [4]. CATCH‐T1D enhances the CHW model by providing much‐needed navigation of specialty diabetes technology needs for minoritized T1D populations. CATCH‐T1D is aimed at using CHWs as trusted resources to boost acceptance of new therapies among minoritized YAs and prompting providers to prescribe technology. Furthermore, by testing CATCH‐T1D in New York, where all Medicaid plans cover CGM and insulin pump devices [18, 25], we address real‐world patient, provider, and system‐level barriers not limited by insurance. This expands the possible utility and dissemination potential of CATCH‐T1D as technology coverage becomes more universal.

The needs addressed in CATCH‐T1D build on previous CHW interventions in Type 2 diabetes, broadening their scope and potentially providing evidence that a diabetes‐specific CHW can positively impact both social care and glycemic outcomes—not only in Type 2 diabetes but also in T1D outcomes by emphasizing T1D technologies [16, 17]. Results from CHW trials in Type 2 diabetes reveal broad improvement in follow‐up and connection to care, as well as HbA1c levels [16, 17]. In addition, noticeable improvements in self‐efficacy and diabetes self‐management have been demonstrated [26].

Our preliminary results show very high recruitment rates, engagement as evidenced by the sheer number of CHW‐participant interactions, and high satisfaction as evidenced by postintervention interviews. Our success is likely attributed to using user‐centered design principles in the development of this intervention [21]. By prioritizing the lived experiences and ideas of the various stakeholders in T1D technology and YA diabetes care, including patients, families and loved ones, pediatric and adult primary and endocrinology providers, social workers, CHWs, and psychologists, our model development process ensured that the eventual intervention aligned with real‐world needs and challenges. By addressing the social factors that directly influence diabetes care, our model supports sustained engagement in both clinical care and diabetes technology use. This approach also fostered a sense of ownership and trust among participants, contributing to higher engagement and more meaningful outcomes.

Care coordination emerged as a critical component in maintaining participant engagement throughout the intervention. Participants reported that CHWs provided a reliable and approachable point of contact who helped them navigate the healthcare system, overcome logistical barriers, and communicate effectively with their providers. The structured CHW workflows—characterized by frequent participant contact and ongoing monitoring for emerging clinical or social needs—ensured timely support and strengthened continuity of care. Another strength of our study was the provider and clinic opt‐in approach, which enhanced program effectiveness by demonstrating providers′ willingness to participate and actively engage with CHWs. Collectively, these elements highlight the multifaceted approach our model adapted to align participant and provider priorities.

Previous clinical trials implementing user‐centered design in its development have found participants reporting more positive experiences and higher trust in researchers and protocols [27, 28]. This could have ripple effects in reporting sensitive information, such as social needs. Our participants attested to feeling more comfortable sharing social needs and asking for the needed resources and assistance due to trust in our study staff and model. This aligns with other findings that reflect building trust is essential to ensuring more accurate, reliable data, ultimately making the study more acceptable and effective in enhancing the capacity to tackle existing challenges in complex health systems [27, 29].

Nevertheless, this study has some anticipated limitations. This is a single‐site study, results from which may not be generalizable to other institutions, especially if they do not have an existing infrastructure to support CHWs. Additionally, our study plans to recruit individuals from Hispanic and non‐Hispanic Black racial/ethnic groups, which does not include other groups who have also been subjected to systemic racism and health inequities, such as Asian/Asian American, Native American, Native Hawaiian, Alaskan Native and Pacific Islanders, or Middle Eastern groups. In addition, new payment models are forthcoming with the announcement of the Medicaid 1115 waiver and other health equity initiatives at the state and federal level, which would need to be integrated into future study implementations but likely will only further bolster the results of this trial if a pay model for CHWs emerges [25]. The innovative design of this model could inform future larger pragmatic trials and support the sustainability of new clinical approaches in T1D care. Future expansions of this study should be aimed at including a wider range of racial/ethnic groups, other forms of diabetes, age groups across the lifespan, and varied healthcare settings to determine the effectiveness and feasibility of this model in these populations.

## 5. Conclusion

CATCH‐T1D is a specialized CHW model that simultaneously addresses social needs and advanced therapeutics for Hispanic and non‐Hispanic Black YA with T1D. In CATCH‐T1D, CHWs offer participants information access to diabetes technology, enhanced shared decision‐making, technology onboarding support, social needs management, offloading of provider burden, and better alignment of patient and provider priorities. The CATCH‐T1D model, designed with the input of YA with T1D, providers, and CHWs, can extend the standard of “team‐based” diabetes care, specifically for underserved communities with T1D. As diabetes technology use becomes more prevalent in Type 2 diabetes, our findings could inform CHW models that might be expanded to other disease processes where populations have both high social needs and pharmacoequity needs.

## Funding

The study is supported by the National Institutes of Health, 10.13039/100000002 (RO1DK132302), and the Helmsley Charitable Trust (G‐2204‐05171).

## Conflicts of Interest

The authors declare no conflicts of interest.

## Data Availability

The data that support the findings of this study are available from the corresponding author upon reasonable request.
